# Microscopy of the Dental Tissues. New Series

**Published:** 1868-10

**Authors:** S. P. Cutler


AMERICAN JOURNAL

OF

DENTAL SCIENCE.

Vol. 11. THIRD SERIES--OCTOBER, 1868. No. 6.

### ARTICLE I.

### *Microscopy of the Dental Tissues. New Series.*

By S. P. Cutler, M. D., A. E. G., D. D. S.,

Histology—Occlusion of Alveolus after Extraction of teeth. Comparatively little is known concerning this process, although similar if not identical to that of repair of injuries of bones in general. The alveolar periosteum or pericemental membrane is thinner than periosteum in general. Whether or not this is a folded sac or double membrane is not yet determined to a certainty. If double, as a general thing, one portion adheres to the fang and the other to the socket; if single, it may all or in part adhere to the one or the other, and if to the fang the alveolus must be denuded in whole or in part. The time requisite for healing or reconstruction of alveolus is equal to that of an injury of any other bone of similar extent in the same individual at the same time; the time varying in differing subjects and different ages of the same individual, and depending in each case on constitutional tendencies or conditions as to health, both physical and mental.

The most rapid belongs to childhood and the most tardy to old age. In the one case the life forces are much more active and energetic, though less resistant. This process may be said to vary from 30 to 50 days though not absolutely complete for more than twice that length of time.

In the time specified I do not claim to be accurate but only approximative. I maintain that when a tooth is extracted, there is a breach made at that point in the organism, in other words an outlet and inlet into the bone or solution of continuity demanding repair. From 10 to 15 days will be required for the establishment of osseous inflammation and exudation of osseous salts. The periosteum covering the maxilla is reflected over the alveolar border down to the bottom of the socket, thence up the fang to the free margin of the gum. This however is only stated as a postulate, and if true this membrane must be double. The first thing that takes place in the extraction of a tooth is the formation of a clot of blood filling the socket, derived chiefly from the apicial artery becoming slightly adherent to the walls, and this is sufficiently vital and organized to resist putrefaction. This plug is a temporary shutter to the breach as a preliminary process and should not be interfered with, as nature rejects it in due time when no longer needed. The first actual change consists in taking down portions of the alveolus and filling up the bottom to a variable extent and lastly the formation of an external table identical to the other bones.

This may be regarded as a healthful or normal process resulting from traumatic inflammation. In the first place there must be colloid cells formed at the bottom of the socket which are subsequently calcified and differentiated into true bone. What carries away the alveolus? It certainly is not oxidation of the lime salts as they are already formed from of lime and phosphoric acid. We must conclude then that an acid is formed in the inflamed part having a stronger affinity for the oxide of lime than the phosphoric acid thereby forming a new salt of lime soluble in the liquor sanguinis, thereby permitting its return to the general circulation and out of the system as waste material, as it could not again be converted into phosphate of lime in the system.

As the lime is thus disintegrated oxidation of the osteine readily takes place.

It is hardly probable that this process is the result of local action resulting from acid formed in the clot removing the lime and systemie oxidation removing the osteine. We conclude then that the lime is removed by an acid and the osteine by oxidation; the lime passping off as a double binary soluble salt and the osteine mainly into carbonic acid water andammonia. At the same timenew bone is being formed in a rapid manner, as nature seems to be in a hurry to repair damages. New bone is formed with an external table quite perfect in the short space of time mentioned above, in fact it is almost as rapid as the healing of flesh after the full commencement of the process. First we have colloid cartilage cells or utricles formed and calcified in the space of two weeks, resulting from inflammatory action in the part, though in slight cases of injury not sufficient to cause much general disturbance, which is wholly different from the slower process of the original formation of bones which is unaccompanied by inflammatory action. Is the removal of the alveolar walls a special process or simply incidental to the healing? We find great difference in the extent of removal of alveolus in different cases even in the same subject, some cases wasting extensively others scarcely any. Before any true calcification can take place, there must be a set of colloid vesicles or utricles formed out ot the blastema carried to the part sufficient for the purpose of forming bone cartillage cells. I may imagine the process of calcification to consist of first a cell deposit of liquid phosphate of lime conveyed to their interior by osmotic force, then, by the absorption of the thinner or watery portions of the lime, the lime becomes dense and adherent to walls of cartillage cells by some unknown process, perhaps resulting from atomic polarity of lime and cartilage molecules, thereby forming a chemical union.

As this hardening process is being carried on, the entire structure of the previous cartilage undergoes differentiation or change and is no longer cartilage but osteine.

This change results in the formation of bone lacunæ and canalieuli which are the reservoirs of nutrient materials or depots of supply for the nutrition of the bone.

This new structure is entirely different from that of the bone cartilage. The cancillated structure of bone is entirely different from that of the cartilage. What precise process takes place has not yet been fully ascertained. The spongy texture of the interior of bones is very different from the previous porous structure of the cartilage, and the thin plates forming the porous structure of bones have the true structure of bone proper. All of these spongy structures of bones must be lined with membrane and that membrane must be continuous into the canaliculi and lacunæ through which absorption by osmosis takes place through these minute tubes, from the blood circulating through the spongy masses. The waste of the lime of bones must, as before stated, be the result of an acid action and the oxidation of osteine. This acid may be the result of oxidation of a portion of the nutrient blastema that is held in reserve in the lacunæ, such as the decomposition of the elements of albumen or starch, or oil, forming carbonic acid and alcohol. By a further oxidation of the alcohol or replacement of two atoms of hydrogen with two of oxygen, we will have acetic acid which will break down the phosphate of lime, reproduce a soluble acetate of lime which would be carried away in solution, and then a replacement of phosphate already there in reserve or carried there in time of need. These ideas are not given as dicta by any means, only as my hypothesis upon a subject so recondite. If there were no nutrition in bones, there could be no life, as life consists in mutability or change or inter-molecular activity which could only take place as purely chemical action in dead structures or where there is no nutrition. I maintain there is no such thing as molecular inertia in the living organism and that life is dependent upon constant mutability or molecular change every where, and that the thermal condition also is wholly dependent on molecular change, which is also a life condition of an organism, and that even the bones to some extent must generate their own caloric.

Every molecular change either in organic or inorganic chemistry results in a thermal disturbance, and life itself is no exception to the rule, that is to say life is based on organic chemistry or chemico-vital forces acting in a specific manner and somewhat different from purely inorganic chemistry,or inorganic chemical action acting on the same materials under different conditions and circumstances. What is here meant by chemical action is mutual action and reaction of matter and force, so called, or atoms of solid matter combined with that something called force and put in motion by it; and the various combinations of different atoms of different elements produce all the different phenomena manifested by chemical action.

It has been discovered that there is going on in living organisms, a continual cell motion or activity in the interior of cells, the true nature of which is not clearly understood as yet.

It may be inferred that each cell, whatever its character, embodies in itself vital or life energies so long as sustained from without with requisite supplies of proper simple or compound elements, namely oxygen and nutrient matter.

It is reasonable to suppose that this cell activity spoken of is molecular activity resulting from chemical or chemico-vital change, resulting in decay and reproduction, or taking down and putting together again, not however out of the same materials, though identical in nature. All the waste matter must be conveyed out and new materials introduced. We know that oxygen enters the tissues with the nutritive elements supplied by food, leaving the capillaries, together passing through their walls, which are perhaps porous or perforated by great numbers of minute apertures too small to be seen even by the microscope, thence in and among the cells which make up or form the tissues, passing freely among them through their interstices and thence entering their chambers through minute pores. We now find free oxygen entering with the nutrient liquid the interior of cells every where at once. What now takes place will be the subject of another article. I will premise by introducing the subject.

In the first step there must be a condensation of oxygen, so as to disturb its latent or combined caloric, so as to convert it into ozone or into an active or combining state (otherwise it is purely inert,) ready to oxidize out the cell wall atoms which are to be replaced.

*To be continued.*

---

